# Surface Chemistry-Dependent Binding Interactions between Kraft Lignin and Polyelectrolyte-Encapsulated Gold Nanoparticles

**DOI:** 10.1021/acsomega.5c07312

**Published:** 2025-10-27

**Authors:** Akinsola A. Oluwaseun, Samuel E. Lohse

**Affiliations:** Department of Chemistry, 3197 Central Washington University, 400 E University Way, Ellensburg, Washington 98926, United States

## Abstract

This study investigated the relationship between gold nanoparticle (AuNP) surface chemistry and the binding affinity of unfractionated kraft lignin to polyelectrolyte-coated AuNPs. Specifically, fluorescence quenching titrations were employed to determine lignin’s binding affinity (*K*_a_) to different charged polymer surfaces displayed on 90 nm citrate-stabilized AuNPs, (poly­(allylamine hydrochloride)) (PAH), polyacrylate (PAA), and poly­(diallyldimethylammonium chloride) (PDADMAC). AuNP–lignin conjugates were characterized by UV–vis absorbance spectroscopy, ζ-potential analysis, and dynamic light scattering (DLS) measurements. The characterization data showed that the size, surface charge and aggregation state of the lignin–AuNP conjugates depended on the original surface chemistry of the AuNP, in conjunction with the lignin concentration. Fluorescence quenching titrations indicated that lignin’s binding affinity for the polyelectrolyte-wrapped AuNPs was significantly higher for PDADMAC-AuNP, while the Cit-, PAH-, and PAA-AuNPs gave statistically indistinguishable affinity constants (*p* < 0.05). *K*_a_ values for PAA-AuNP, Cit-AuNP, PAH-AuNP, and PDADMAC-AuNP were determined to be 87 ± 8 nM^–1^, 92 ± 11 nM^–1^, 107 ± 13 nM^–1^, and 240 ± 13 nM^–1^, respectively. This fluorescence data indicates that electrostatic interactions are not the primary driving force in lignin adsorption to these AuNP surfaces. Instead, van der Waals forces (such as hydrophobic interactions and hydrogen bonding) are likely more important in mediating lignin–AuNP adsorption on this length scale. Understanding how the surface chemistry of polymers mounted on nanoscale surfaces impacts lignin adsorption will inform the process of eco-corona formation on polymer-coated nanoscale surfaces.

## Graphical abstract

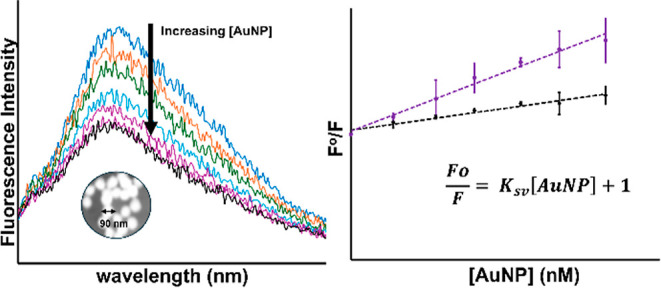

## Introduction

As a result of their unique size regime and size-dependent properties, the use of engineered nanomaterial (ENM)-enabled consumer products has increased significantly in the past decades.^1–10^ These ENMs have found multiple applications in health care, water purification, energy, agriculture, packaging, and personal care products.^1,3,5,6,9^ As a trade-off, however, ENM’s increasing accumulation in the environment (primarily due to their release at the end of a nanoenabled product’s lifetime) has made these emerging materials contaminants a looming concern.^3,4,7^ Simultaneously, it has now been fully recognized that micro- and nanoplastics (formed by the erosion of macroscale plastics) have already permeated air, soil and water across the globe.^1,2,4,7,10^ Their unique size regime (1–100 nm diameter) and size-dependent properties place both ENMs and nanoplastics in an emerging class of nanoscale contaminants whose fate, transport and toxicity cannot easily be predicted based solely on their chemical composition.^3,4,7–9^ Recent reports, highlighting the ability of nanoplastics to cross the blood–brain barrier, have further raised concern about the ubiquitous nature of nanoscale contaminants in the environment.^11^ In addition to the inherent (and poorly understood) toxicity of these nanoscale contaminants, the potential for these material contaminants to concentrate other molecular pollutants on their surfaces raises concern that nano contaminants can act as carriers (or vectors) for molecular contaminants.^3,12–17^ In order to understand the true risk of these emerging contaminants, the environmental fate, transport, and toxicity of these materials must be formally linked to their physiochemical properties (size, shape, composition, and surface chemistry).^3,13^ This fate and transport analysis is, however, complicated by the fact that as soon as a nano contaminant enters an environmental medium, the “synthetic identity” of the nanomaterial is altered by the process of eco-corona formation, giving the nanomaterial a new “biological identity”.^12–14^

As these nano contaminants enter the natural environment, natural organic matter (NOM, *e.g.*, lignin, humic and fulvic acids), proteins, lipids, polysaccharides, pollutants, etc., adsorb to the ENM surface.^18–20^ These biopolymers and organic molecules form a new adsorbed surface layer around the material, recently dubbed an “eco-corona” (in analogy to the widely recognized biomolecular corona that forms on nanomaterials in serum).^12–14^ The eco-corona modifies the surface and chemical properties of the ENMs, significantly influencing their migration, aggregation, biodistribution, and toxic effects.^12–14^ For instance, humic acid adsorption onto the surface of polyethylene and polystyrene microplastics has been shown to reduce the affinity of pharmaceutical contaminants for their adsorption sites on the polymer surface.^21^ The eco-corona therefore controls the new environmental identity of the NP, dictating (for better or worse) its interactions with its surroundings.^10,12–15^ Despite the importance of the eco-corona in controlling the environmental behavior of ENMs, the chemistry by which the corona forms remains incompletely understood, largely due to the high complexity of the factors (environmental medium, incubation time, surface chemistry of the ENM, presence of contaminants, etc.) that can influence the composition and thickness of the corona.^13^

Although the relative complexity of eco-coronas can be daunting, investigating the adsorption affinity of specific corona-forming components (like NOM) for the surfaces of ENMs is a feasible starting point to understand the connection between ENM physiochemical properties and corona composition.^13,22^ Understanding how the ENM is seen by NOM, lipopolysaccharides, proteins, etc. in freshwater is therefore an essential step in a detailed understanding of the ecological transformations of these ENMs.^13^ Investigating the binding of individual corona components to NP surfaces has been previously employed to understand the individual chemical interactions through which biomolecular coronas (particularly protein coronas) form on the surface of nanotherapeutics.^22–29^ The affinity of specific biomolecules for NP surfaces can be quantified using a variety of instrumental techniques, with the strength of binding typically being quantified as the affinity constant (*K*_a_) of the biomolecule for the surface of the particle.^23,25,26^ These instrumental techniques include fluorimetry,^25,26^ UV–vis absorbance spectroscopy,^23–26^ dynamic light scattering,^26^ affinity capillary electrophoresis^25^ and isothermal titration calorimetry.^25–29^ Among these approaches, fluorescence quenching titration (FQT) is one of the most convenient techniques to investigate the binding interactions between a corona-former that fluoresces and a molecule that quenches the intensity of the fluorescing molecule (e.g., a nanoparticle).^25,26^ In the context of FQT, the affinity constant of the corona-forming molecule for the nanoscale surface is determined by fitting the raw fluorescence data to the Stern–Volmer relationship, and extracting the affinity constant from the slope of the plot.^25,26,30^

Lignin, a type of NOM derived from the lignocellulose biomass of plants, is recognized as one of the most abundant biopolymers on earth.^31–37^ Lignin has gained special recognition as a potential renewable feedstock for aromatics.^31–37^ Lignin’s chemical structure is varied, depending on its exact source and isolation method, but lignin’s structure is generally marked by a network of branched aromatic moieties, displaying aliphatic and phenolic hydroxyl groups, methoxy groups, and carboxylic acid groups on the side chains.^36^ Lignin is generally considered to be composed of monomer units derived from three hydroxycinnamic alcohols (*p*-coumaric, coniferyl, and sinapyl alcohol).^36^ These functional groups enable various intermolecular interactions, including Coulombic attraction, hydrogen bonding, van der Waals forces, and hydrophobic interactions between lignin and ENM surfaces.^31–37^ Previous studies have shown lignin’s substantial capacity to exhibit significant adsorption to a wide range of pollutants and contaminants, due to its high surface area and available hydrophilic and hydrophobic functional groups. Recent studies have further indicated that lignin may also possess the ability to adsorb microplastics and metal nanoparticles such as gold (Au) and silver (Ag) from the environment, thereby facilitating their removal or sequestration.^33^ One of the most useful properties of lignin (as opposed to other classes of NOM) for this study is lignin’s inherent fluorescence.^38^ Although possessed of a low quantum yield compared to synthetic fluorophores (φ ∼ 0.10), lignin’s luminescence has been used to enable a number of specific fluorescence applications, including ratiometric pH sensing.^38^ This makes lignin a very useful class of model NOM for studying the fundamental adsorption events that lead to the formation of the eco-corona.

Many ENMs and nanoplastics are complex chemical systems whose detection and characterization in the environment are challenging using traditional instrumental approaches.^3–5,13^ Among the ENM probes available to study the thermodynamics and kinetics of NOM-NP binding interactions, AuNPs are one of the most convenient and versatile.^39–43^ Gold nanoparticles are a particularly useful ENM model because their core shape and dimensions can easily be synthetically controlled, and their surfaces can be conveniently modified to display thiol monolayers, functionalized silica shells, or nanoscale polymer surfaces.^39,42^ Furthermore, AuNPs can be easily tracked within environmental media (compared to other nanoparticles), due to their superior stability against oxidation, low background levels and size-dependent optical properties.^39,42,44^ Most crucially for corona-forming studies, however, the AuNPs possess size- and shape-dependent surface plasmon resonance (SPR) absorbance and scattering behaviors that are sensitive to the AuNP’s local environment.^39,40^ These properties allow the fate and transport of AuNPs to be closely monitored and correlated with their physiochemical properties within various environmental media.^42,44^ In FQT experiments, AuNPs are often used as fluorescence quenchers, making them a viable substrate to measure the affinity of fluorescent NOM (lignin) to nanoscale surfaces.^25,26^

The goal of this study was to measure the affinity of kraft lignin for AuNPs displaying different polyelectrolytes using FQT and characterize the size, surface charge and aggregation state of the resulting lignin–AuNP conjugates. To achieve this, 90 nm AuNPs stabilized with citrate and three polyelectrolytes were prepared using established synthetic methods.^45,46^ This produced a library of 90 nm AuNPs displaying four distinct surface chemistries (Cit-, PAH-, PAA- and PDADMAC-AuNPs, Figure 1). The AuNP library therefore included polyelectrolyte-wrapped AuNPs displaying both positively and negatively charged functional groups, and functional groups whose charges both are pH-sensitive (Cit, PAH, and PAA) and pH-insensitive (PDADMAC). The 90 nm AuNP probes were incubated with aqueous lignin solutions at 25 °C, and then the resulting lignin–AuNP conjugates were characterized using UV–vis absorbance spectroscopy, DLS, and ζ-potential analysis. Once we verified that the lignin had adsorbed successfully to the AuNP surface, the affinity constant of lignin for each AuNP surface was determined using FQT.

**Figure 1:**
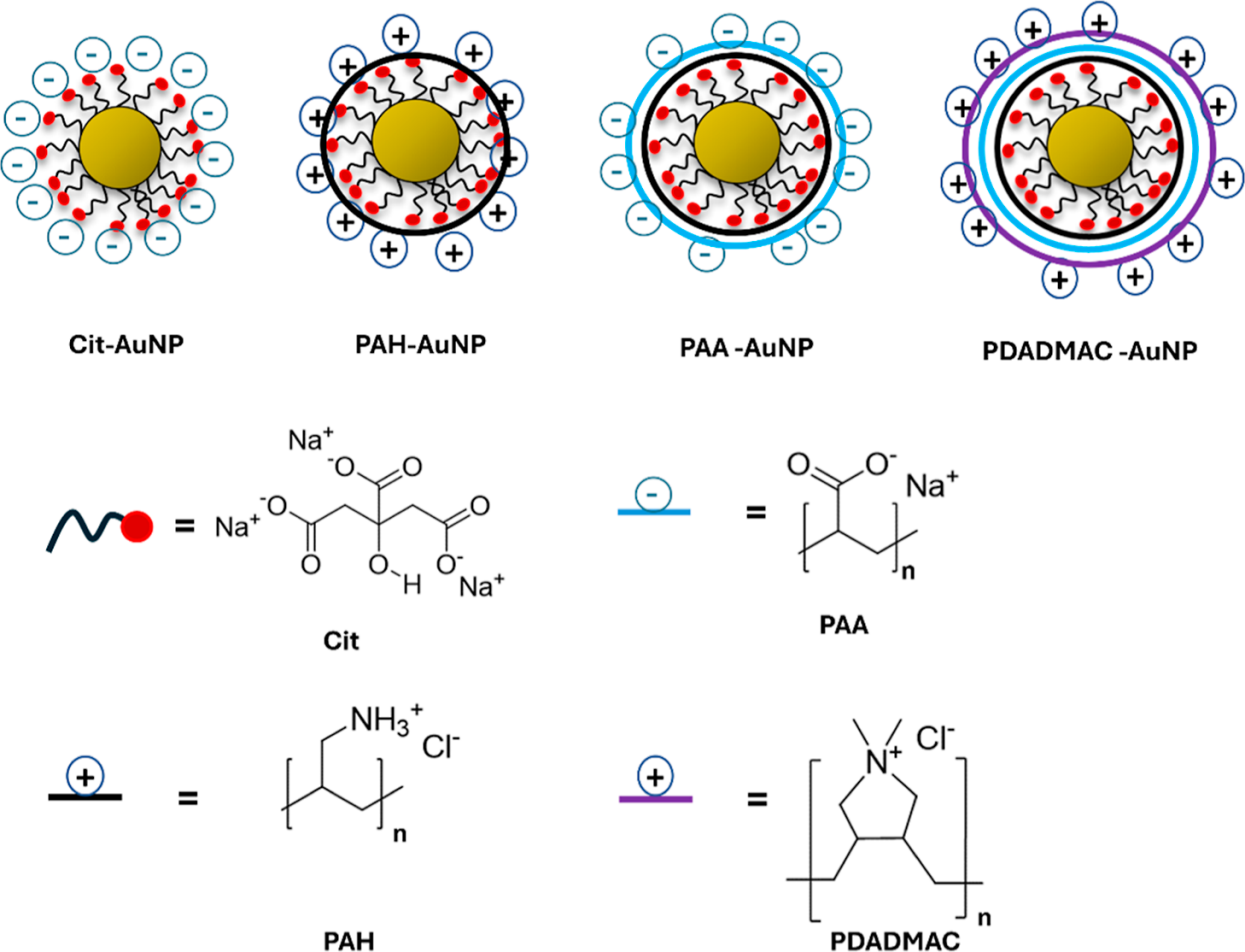
Schematic representation of the 90 nm citrate (Cit) and polyelectrolyte (PE)—coated AuNPs used in this study.

## Results

### AuNP Size and Surface Chemistry Characterization

Prior to polyelectrolyte coating, gold nanoparticles were characterized using UV–vis absorbance spectroscopy, DLS, and SEM to confirm their size. Figure 2 shows the SEM images of the purified Cit-AuNP dispersion and the statistical analysis of the particle core sizes using ImageJ software (*n* = 188). The resulting data demonstrated that the synthesized Cit-AuNPs are isotropic with an average core diameter of approximately 84 ± 10 nm. The UV–visible absorption spectrum of the purified Cit-AuNP dispersion (Figure 2C) exhibited the maximum surface plasmon resonance absorption (SPR) at a wavelength of 554 nm (λ_spr_ = 554 nm). The plasmon absorbance corresponds to an average core diameter (*d*_core_) of approximately 86 ± 3 nm.^43^ AuNP size was further confirmed by measuring the particles’ hydrodynamic diameter (*D*_h_) using DLS. Unlike the core diameter measured by SEM and absorbance spectroscopy, the hydrodynamic diameter represents the overall size of nanoparticle, including the thickness of the capping agent layer, and any closely associated solvent molecules surrounding it.^26,27^ Figure 2D displays the distribution of the AuNP’s *D*_h_ weighted for intensity (Figure S1, Supporting Information shows a comparison of the DLS weighted for both intensity and particle volume across all the AuNP surface chemistries studied). From the DLS data, the *D*_h_ of the Cit-AuNPs was determined to be 88 ± 6 nm. The composite picture of the AuNP size characterization data (SEM, UV–vis and DLS) suggests that the size of the Cit-AuNP is consistent with UV–vis and SEM measurements, and the overall nanoparticle size is close to 90 nm.

**Figure 2:**
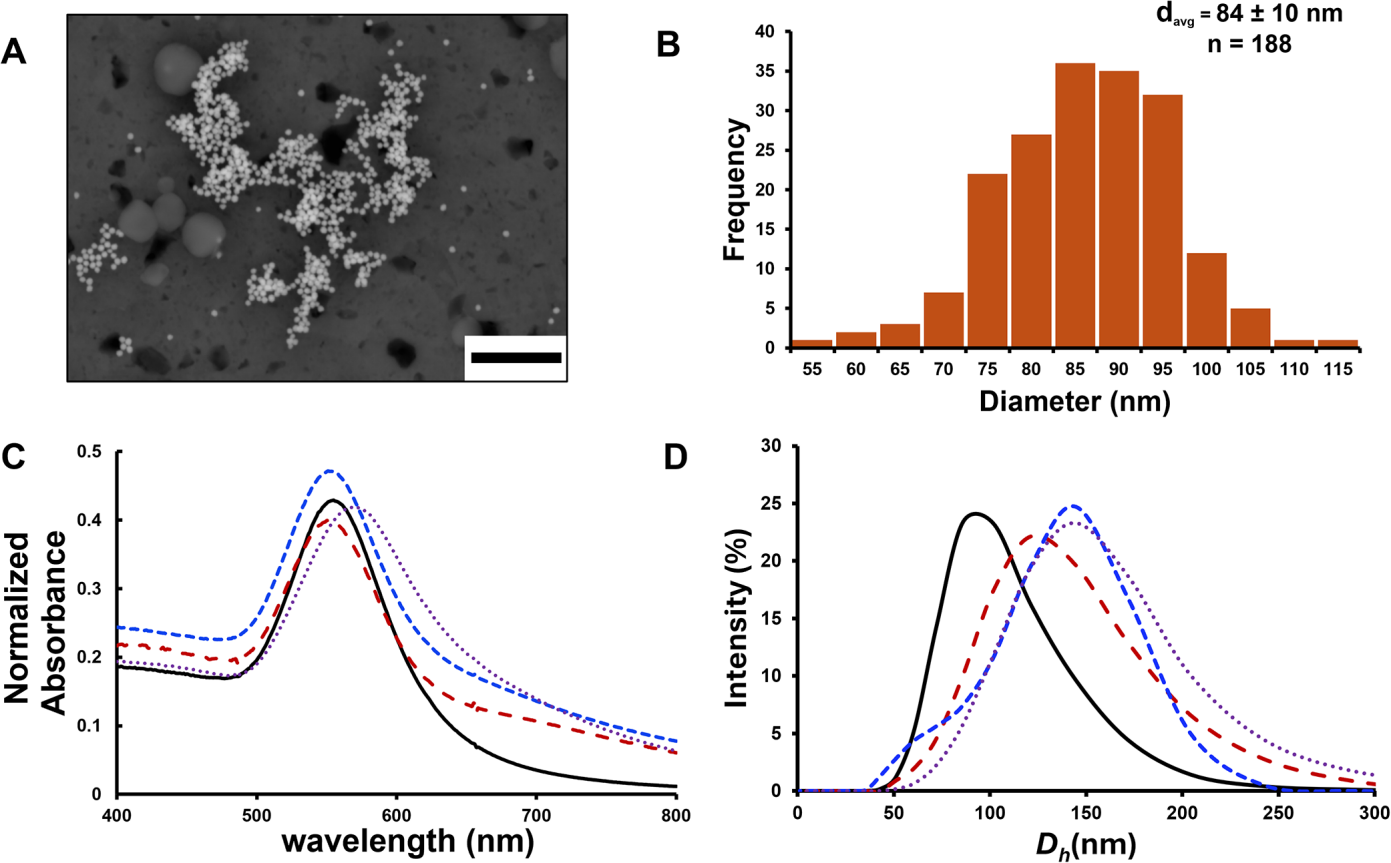
(A) Scanning electron micrograph of the as-synthesized AuNPs. The scale bar is 1 μm. (B) Histogram of particle core sizes from the SEM data, showing the statistical distribution of the individual particle sizes. (C) UV–visible absorbance spectra of aqueous Cit-(black, solid line), PAH-(red dashed line), PAA-(blue dashed line), and PDADMAC-(purple dotted line) AuNP dispersions. Absorbance spectra obtained at pH = 7.4, [AuNP] = 0.0033 nM. (D) Dynamic light scattering intensity particle size distribution plot indicating the distribution of hydrodynamic diameters. DLS measurements obtained at pH = 7.4 (1 mM bicarbonate buffer), [AuNP] = 0.0033 nM.

DLS, ζ-potential analysis, UV–vis, and FTIR spectroscopy were used in concert to verify the functionalization of the AuNPs with each successive layer of polyelectrolyte on the AuNP surface (Figure 2C,D, and Table 1). DLS analysis indicates that each PE wrapping step adds to the overall thickness of the particle with the next layer of polyelectrolyte successively contributing to the *D*_h_, generating a new hydrodynamic diameter larger than the previous particle diameter (an increase of about 12 nm per layer). The ζ-potential measurement further confirms that the surface charge of the particle changes predictably after each layer of PE, corresponding to the charge of the polyelectrolyte displayed on the outer layer. Wrapping with PAH and PDADMAC leads to polyelectrolyte-wrapped AuNPs with a positive charge, while the Cit-AuNPs and PAA-AuNPs display a net negative charge. ATR-FTIR spectroscopy confirmed the presence of the desired functional groups on each particle following layer-by-layer PE assembly (Figure S2, Supporting Information).

**Table 1:** Hydrodynamic Diameter and ζ-Potential of the Functionalized AuNPs Pre- and Post-Lignin Exposure

	*D*_h_ (nm)	ζ-potential (mV)	*D*_h_ (nm)	ζ-potential (mV)
AuNP surface chemistry	pre-lignin exposure^a^		post-lignin exposure	
Cit	88 ± 6	–42 ± 5	150 ± 31	–24 ± 3
PAH	101 ± 6	+44 ± 5	135 ± 17	–43 ± 17
PAA	112 ± 17	–88 ± 8	100 ± 11	+32 ± 80
PDADMAC	125 ± 8	+59 ± 9	122 ± 22	–37 ± 3

a Pre-lignin exposure conditions are Milli-Q water at pH = 7.4 (bicarbonate buffer), [AuNP] = 0.0033 nM. Post-lignin exposure conditions are AuNP–lignin conjugates prepared in 0.2 mg/mL lignin solution at pH = 7.4 (1 mM bicarbonate buffer), [AuNP] = 0.0033 nM.

### AuNP–Lignin Interaction and Characterization of the Eco-corona

As part of the preparation for the fluorescence quenching titration study, we characterized the lignin and the lignin–AuNP conjugates that form in solution by UV–vis absorbance spectroscopy, DLS and ζ-Potential analysis. These measurements were obtained over a range of lignin concentrations (0.0–0.2 mg/mL) and using the highest gold nanoparticle concentration that we used in the FQT studies ([AuNP] = 0.0033 nM) in a bicarbonate-buffered solution (pH = 7.4). These measurements indicated that (under these specific conditions) the interactions of lignin with AuNP surfaces are potentially complex, leading to the formation of single-component eco-coronas that are surface-chemistry-dependent. However, the structure of the lignin–AuNP conjugate is not necessarily restricted to the direct adsorption of lignin to the AuNP surface. DLS analysis revealed that the unfractionated kraft lignin used in this study is inherently polydisperse, with multiple peaks in the size distribution occurring between 102 and 6450 nm, indicating that less than 25% of the lignin molecules have hydrodynamic diameters below 200 nm, and the majority of the lignin polymer exists in clusters significantly larger than the PE-coated AuNPs themselves (Figure S3, Supporting Information). ζ-Potential analysis of the same lignin solution indicated that the lignin itself has a meaningfully negative surface charge, but significant variability (−29.6 ± 15 mV). When the original AuNPs are incubated in a 0.2 mg/mL aqueous solution of lignin, the surface charge of the AuNPs generally shifts to a negative surface charge similar to the lignin itself (Table 1, Figure 3), which would be consistent with lignin overcoating the AuNP surface. However, the PAA-AuNPs’ surface charge, postlignin exposure, shifts to near-neutral, even though the PAA-AuNPs and the lignin itself both have overall negative surface charges.

**Figure 3:**
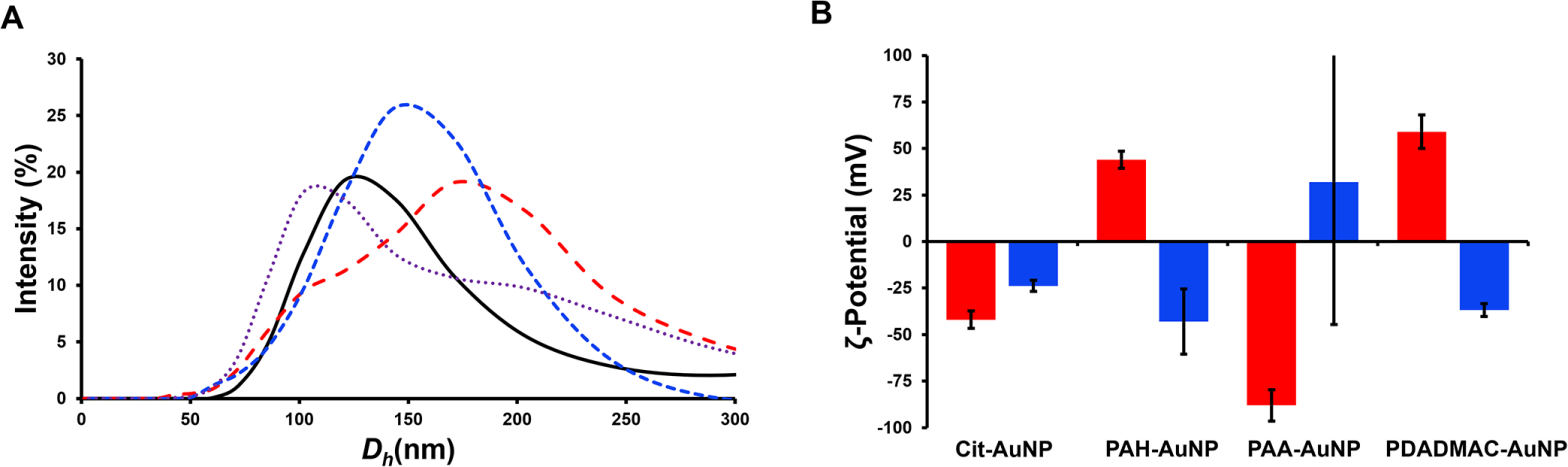
(A) Dynamic light scattering intensity particle size distribution plot indicating the distribution of hydrodynamic diameters for lignin–AuNP conjugates. Cit-(black, solid line), PAH-(red dashed line), PAA-(blue dashed line), and PDADMAC-(purple dotted line) AuNP dispersions. (B) ζ-Potential analysis of functionalized AuNPs pre- and postlignin incubation. DLS/ζ-potential measurements obtained at pH = 7.4 (1 mM bicarbonate buffer), [AuNP] = 0.0033 nM. Red: pre-lignin exposure. Blue: post-lignin exposure.

Close analysis of the DLS data also indicates that lignin binding to the surface of the AuNPs does not necessarily result in an eco-corona that is analogous the traditionally understood model of the protein corona. Instead, the binding of the lignin to the AuNP surface results in lignin–AuNP conjugates that have surface-chemistry-dependent sizes. The lignin–AuNP conjugates for the Cit-AuNPs and PAH-AuNPs have *D*_h_ values larger than the original *D*_h_ value for the particles (Table 1, Figure S4), but the PAA-AuNPs and PDADMAC-AuNPs have *D*_h_ values that are statistically indistinguishable from those of the original AuNPs (two-tailed *t*-test, *p* < 0.05). While this lack of change in particle size could be attributable to conformational reorganization of the PE layers upon lignin binding, the size intensity plots of the DLS data (Figures 3A, S4, S5, and Table S1) actually indicate that the lignin–AuNP conjugates may be more polydisperse in size than original AuNPs. For instance, the PAH- and PDADMAC AuNP size distributions actually skew slightly bimodal following lignin exposure, while the Cit- and PAA-AuNP size distributions do not. Although the Cit-AuNP–lignin conjugates show a low-intensity, but detectable peak around 500 nm. The PAH-AuNP–lignin and PDADMAC-AuNP–lignin conjugates show two broad peaks at ∼110 nm and ∼200 nm. However, the intensity of the 110 nm peak is higher in the PDADMAC sample, and the intensity of the 200 nm peak is higher in the PAH-AuNP sample.

The UV–vis absorbance spectra of the AuNPs (postlignin incubation) suggest that PDADMAC-AuNP particle aggregation occurs following exposure to lignin at lignin concentrations of less than 0.2 mg/mL, with the SPR absorbance of the PDADMAC-AuNPs broadening and the baseline of the spectrum raising (due to plasmonic coupling), following lignin exposure (Figure S6). This plasmonic coupling would indicate that the PDADMAC-coated AuNPs undergo some hetero aggregation with the lignin during incubation (either under [AuNP] > 0.0033 nM or below 0.2 mg/mL lignin), while the Cit-, PAH-, and PAA-AuNPs do not appear to be aggregating upon lignin exposure. This type of aggregation behavior was not observed at PDADMAC-AuNP concentrations less than 0.0033 nM, or at lignin concentrations greater than 0.1 mg/mL and so no [AuNP] > 0.0033 nM were used in the subsequent fluorescence quenching titration studies to ensure that the adsorption of lignin to particle surfaces was studied in the absence of AuNP hetero aggregation as a competing process. At the [AuNP] and [lignin] concentrations used in the FQT studies, the PDADMAC-AuNPs appear to remain well-dispersed, with no obvious competing aggregation process (Figure S7, Supporting Information). Complete absence of hetero aggregation cannot be fully ruled out, based on the small shift in the *D*_h_ for the PAH- and PDADMAC- toward bimodal size distribution upon lignin exposure. The Cit-AuNPs also show the emergence of a broad, shallow DLS peak at ∼500 nm (Figure S5), which could also be consistent with the Cit-AuNPs undergoing minor aggregation upon lignin adsorption, or a small fraction of the Cit-AuNPs binding to larger lignin polymers.

### Fluorescence Quenching Measurement of Lignin–AuNP Affinity Constants (*K*_a_)

After purification of the PE-AuNPs and characterization of their surface chemistries, affinity constants (*K*_a_) for lignin binding to each nanoscale polyelectrolyte surface was achieved through a fluorescence quenching titration experiment. In this experiment, varying concentrations of AuNPs ranging from 0 to 0.0033 nM were exposed to lignin at a constant concentration (0.2 mg/mL lignin) in bicarbonate-buffered solution (pH = 7.4) and incubated at room temperature (25 °C) in the dark for 60 min. After incubation, the lignin fluorescence intensity in the absence of the quencher (in this case, the AuNP), *F*_0_, and the fluorescence intensity in the presence of the quencher, *F*, at λ_em_ = 365 nm were measured. The resulting fluorescence ratio (*F*_0_ */F*) was then plotted against the [AuNP] to construct a Stern–Volmer plot, which was used to determine the value of *K*_a_. The experiment was performed in triplicate (*n* = 3). A lignin concentration of 0.2 mg/mL was chosen for these studies because a maximum amount of lignin fluorescence (∼7000 cps, λ_em_ = 365 nm) was observed at this concentration; higher and lower lignin concentrations produced reduced overall fluorescence under the same conditions.

If the lignin and the AuNP bind to form a stable complex (static quenching), the affinity constant (*K*_a_) between the two species can be obtained directly from the slope of the Stern–Volmer plot (i.e., *K*_sv_ = *K*_a_).^25–27^ This fluorescence quenching technique has been previously employed to determine the strength of absorption between serum proteins (e.g., albumin) and AuNPs of different sizes and surface chemistries. We found that our data supported a static quenching interaction between lignin and the AuNP surface in three ways. First, we observed that when at least two of the AuNP surface chemistries that we studied were exposed to increasing lignin concentrations, the λ_spr_ of the AuNP dispersion underwent a shift, consistent with the lignin binding to the AuNP surface, and changing the dielectric environment around the AuNP (Figure S6). Second, the DLS and ζ-Potential analysis data of the AuNPs (pre- vs postlignin exposure, Figure 3 and Table 1) indicated that the AuNPs generally increase show an increase in their *D*_h_ after lignin exposure and show a surface charge that would be consistent with lignin overcoating the original polyelectrolyte surface of the particle. Third, in the construction of the Stern–Volmer plots from the fluorescence quenching data, we observe linear increases in the *F*_0_ */F* ratio with [AuNP] for each AuNP surface chemistry studied. All these data are consistent with the lignin and AuNPs forming a stable complex upon binding (a static quenching situation). Therefore, the relevant lignin–AuNP *K*_a_ values can be obtained from the slopes of their respective Stern–Volmer plots.

In the fluorescence quenching titration experiments, a clear trend of increasing AuNP concentrations leading to a gradual decrease in the fluorescence intensities of the lignin molecules was observed (Figures 4, S8 and S9, Supporting Information). Saturation of fluorescence quenching was then observed at a [AuNP] ∼ 0.010 nM. As discussed above, the slope of the Stern–Volmer plot reflects the affinity of lignin for the different AuNP surfaces. Significantly, when the slope of the plot is large, it indicates a stronger adsorption interaction between lignin and the AuNP, while a smaller slope indicates a weaker adsorption interaction. Of the AuNP surface chemistries examined in this study, three of the AuNP surfaces (Cit, PAH, and PAA) show similar affinity for the lignin (similar slopes in the Stern–Volmer plot), while one surface chemistry (PDADMAC) shows a significantly greater affinity for the lignin. The *K*_a_ values obtained for each AuNP surface chemistry are listed in Table 2. In the fluorescence quenching titrations, PDADMAC-AuNPs (*K*_a_ = 240 ± 13 nM^–1^) demonstrated the highest affinity for lignin, significantly stronger (two-tailed *t*-test, *p* < 0.05) from the other surface chemistries. Cit-AuNPs, PAH-AuNPs, and PAA-AuNPs showed statistically indistinguishable affinity for lignin adsorption, despite the different charges and functional groups displayed by these AuNP surfaces.^30^

**Figure 4:**
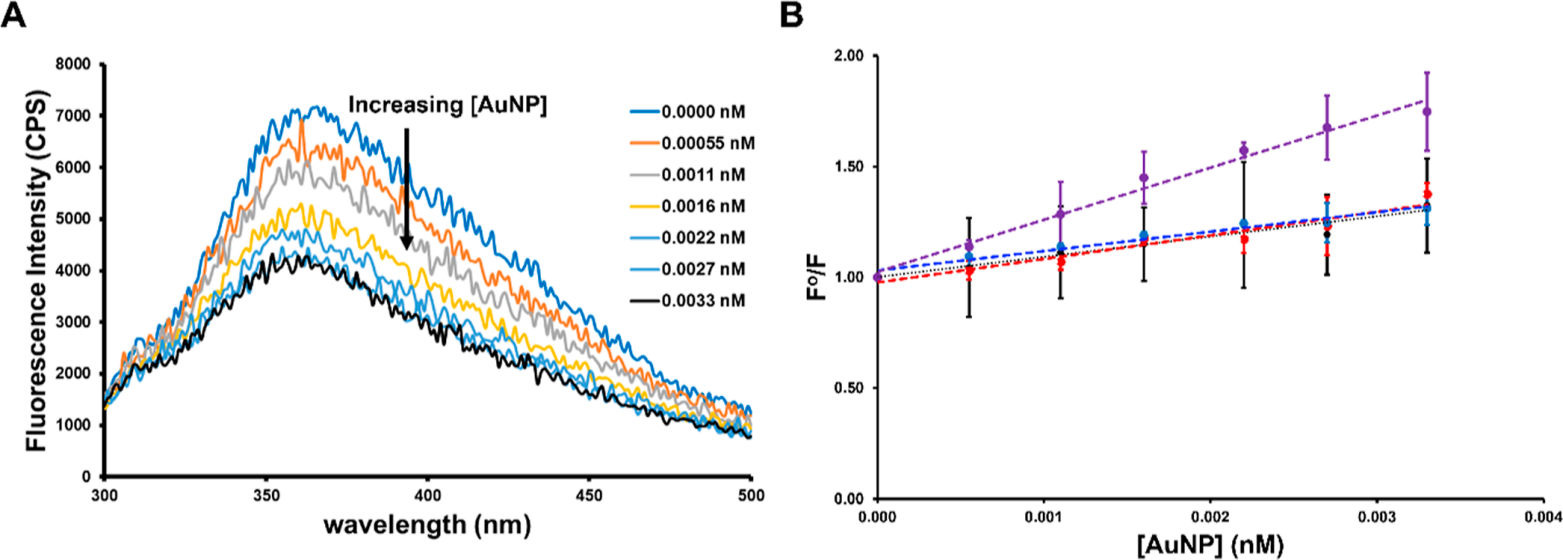
(A) Example fluorescence quenching spectra for 0.2 mg/mL lignin incubated with PDADMAC-AuNPs at increasing [AuNP] (0–0.0033 nM). AuNP dispersions were incubated with lignin for 60 min in the dark at 25 °C, pH = 7.4 (1 mM bicarbonate buffer). (B) Stacked Stern–Volmer plots for Cit-AuNPs (black), PAH-AuNPs (red), PAA-AuNPs (blue), and PDADMAC-AuNPs (purple). Error bars represent standard deviations for replicate trials (*n* = 3). In some cases, the error bars do not extend past the width of the original data point.

**Table 2:** Affinity Constants (*K*_a_) Obtained from Fluorescence Quenching Titration Experiments^a^

AuNP surface chemistry	*K*_a_ (nM^–1^)
Citrate	92 ± 11
PAH	107 ± 13
PAA	87 ± 8
PDADMAC	240 ± 13

a *K*_a_ values obtained from AuNP–lignin conjugates prepared in 0.2 mg/mL lignin solution at pH = 7.4 (1 mM bicarbonate buffer). λ_em_ = 365 nm.

## Discussion

### Lignin–AuNP Conjugate Size Characterization

The inherent polydispersity of lignin can significantly impact its adsorption of chemistry on the surface of more monodisperse nanomaterials.^47–50^ Since lignin is a potential component of nanomaterials’ eco-coronas that can occur in a number of different environmental media, it is important to recognize that lignin’s inherent polydispersity (in size, structure, and composition) may lead to the formation of poly disperse lignin–AuNP conjugates whenever lignin adsorption occurs (particularly in unfractionated lignin samples). It is also important to recognize that lignin polymers are large enough (unlike proteins and biomolecules) to bind 1:1 with 90 nm AuNPs (and saturate the surface of the particle with a single polymer), or to hetero aggregate (and potentially cross-link) multiple AuNPs during the eco-corona formation process.

Our lignin–AuNP conjugate characterization described here indicates (through a combination of UV–vis absorbance spectroscopy and DLS analysis) that when the 90 nm AuNPs are exposed to the unfractionated kraft lignin, each AuNP forms a relatively unique AuNP–lignin conjugate size based on the surface chemistry of the AuNP (Figure 3, Table 1). For instance, when 90 nm Cit-AuNPs and PAH-AuNPs are exposed to the lignin solution (0.2 mg/mL lignin), the hydrodynamic diameter of the particle increases by at least 40 nm. However, the *D*_h_ values obtained for 90 nm PAA- and PDADMAC-AuNPs are not significantly different than the *D*_h_ of these AuNPs prelignin exposure (two-tailed *t*-test, *p* < 0.05, Figure S4). This should not be interpreted as lignin failing to adsorb to the PAA- and PDADMAC-AuNP surfaces, however, as the PAA- and PDADMAC-AuNPs both show a clear change in the *D*_h_ distribution and their ζ-potential following incubation with lignin. Further evidence of the lignin adsorbing to the PDADMAC-AuNPs can be seen in the change in the SPR λ_max_ upon incubation with lignin (UV–vis absorbance spectra). Interestingly, the PAH- and PDADMAC-AuNPs (both particle types with positive surface charges) show a bimodal *D*_h_ size distribution following lignin exposure. Taken together, the UV–vis absorbance data, DLS and ζ-potential data would seem to suggest that the AuNPs form lignin–AuNP conjugates that are somewhat polydisperse in size following their incubation with lignin (at least under the conditions studied).

It is also particularly important to note that the lignin–AuNP conjugates we are characterizing here were being formed at relatively high lignin/AuNP concentration ratios, with lignin as the only component of our model corona. In a typical environmental medium, both the lignin concentration and gold nanoparticle concentrations will generally be lower than the concentrations used in our study, and other corona forming species (e.g., lipopolysaccharides, other classes of NOM, etc.) will likely be more prevalent. This physiochemical characterization of lignin–AuNP conjugates (while relevant to our subsequent binding constant study) likely has limited application to lignin coating of ENMs in many environmental media. The polydispersity in the lignin–AuNP conjugates could arise due to three likely causes (although other explanations may also be feasible), and all three scenarios may operate synergistically in the adsorption of lignin to the AuNP surface.^13^ First, the polydispersity of the lignin polymers leads to the polydispersity of the lignin–AuNP conjugates. Based on our DLS analysis of the kraft lignin itself (Figure S3), the lignin itself is multimodal in size distribution, with *D*_h_ maxima occurring at <500 nm, ∼2000 nm, and >5000 nm. Since all the lignin–AuNP conjugates have *D*_h_ values significantly below 200 nm, it would seem to suggest that the 90 nm AuNPs adsorb the smaller (*D*_h_ < 500 nm) lignin polymers preferentially, but the polydispersity inherent in the lignin leads to corresponding polydispersity in the size of the lignin–AuNP conjugates. Second, as the PE layers on the AuNP surface get thicker (as more PE layers are added to the surface), the lignin may be able to intercalate into the PE layers, and could potentially mix with, displace or deform the PE layers. PE-coated AuNRs have previously been shown to “loosen” the packing of the PE layers on the AuNR surface (in response to photothermal stimuli and pH effects),^51–53^ so it is possible that lignin could merge with the existing PE layers during corona formation, rather than simply adsorbing over the top of the PE layer. Third, the kraft lignin contains large enough lignin polymers that some degree of particle-lignin hetero aggregation (through lignin cross-linking of AuNPs) could be a competing process to monodisperse corona formation, leading to polydispersity in AuNP–lignin conjugates through the formation of AuNP–lignin dimers, trimers, etc. Some evidence can be seen in the DLS data for this process, and the PAH- and PDADMAC-AuNPs show a second *D*_h_ maximum at approximately twice the primary AuNP size (∼200 nm), and plasmonic coupling is observed in the UV–vis absorbance spectrum of the PDADMAC-AuNPs at [AuNP] > 0.0033 nM or [lignin] < 0.1 mg/mL. The plasmonic coupling in the UV–vis absorbance spectra would provide strong evidence that the PDADMAC-AuNPs do indeed hetero aggregate with the kraft lignin under some concentration conditions (but minimal detectable hetero aggregation occurs in concentration range at which the FQT adsorption studies were performed). In contrast, the UV–vis absorbance spectra of the PAH-AuNPs does not show plasmonic coupling at any concentration range studied, and it is not clear from the data whether the PAH-AuNPs would participate in hetero aggregation with the lignin polymers. Lastly, it is important to note that the PDADMAC polymer is the largest and most polydisperse polyelectrolyte used in this study (average *M*_w_ < 100, 000), and the size of the PE layer itself may have some bearing on how the PDADMAC-AuNPs interact with the lignin polymers.

### Lignin Adsorption Affinity as a Function of Surface Chemistry

The fluorescence quenching phenomenon provides valuable insights into the binding interactions of lignin to the surface of AuNPs, such as affinity strength and (in some cases) binding mode.^25,26,48–50^ The primary objective of our FQT experiment was to quantify the adsorption affinity of lignin to the surfaces of the Cit, PAH, PAA, and PDADMAC-AuNPs. The raw data obtained revealed a clear trend of fluorescence loss in the presence of increasing AuNP concentrations (Figure S8). Along with the DLS and UV–vis absorbance data obtained on the lignin–AuNP conjugates, the loss of fluorescence confirms that lignin binds with each of the AuNPs in the study to form a lignin–AuNP conjugate, and the lignin’s fluorescence is therefore lost through static quenching. The higher the slope of the Stern–Volmer plot, the larger the affinity constant *K*_a_, and the stronger the adsorption interaction between lignin and the AuNP surface. The surface charge and functional groups of each polyelectrolyte displayed on the surface of the AuNP should play a vital role in mediating their adsorption interaction with lignin. Since the lignin carries a mild negative charge (ζ-potential analysis), going into this study, our expectation was that the charges of the PEs coating the AuNP surfaces would mediate the binding of the lignin through Coulombic attraction and repulsion. Previous quartz-crystal microbalance with dissipation monitoring (QCM-D) experiments in which carboxylate-modified lignin was allowed to adsorb to PDADMAC thin films supports the idea that electrostatics were the primary driving force in mediating lignin-PDADMAC binding events (for modified lignin polymers).^54–56^

Surprisingly, of the four AuNP surface chemistries studied, only one (PDADMAC) was found to have a significantly different affinity for the unfractionated kraft lignin. The *K*_a_ value obtained for PAH-AuNP was not significantly different from the PAA-AuNP and Cit-AuNPs *K*_a_ values (two-tailed *t*-test, *p* < 0.05). PDADMAC-AuNPs (*K*_a_ = 240 ± 13 nM^–1^) demonstrated the highest affinity constant for lignin. The fact that the affinity of lignin for 90 nm PAH-AuNPs is indistinguishable under these conditions from the negatively charged Cit-AuNPs and PAA-AuNPs suggests that electrostatics are not the determining factor in lignin adsorption under these conditions. Instead, since Cit, PAA, and PAH are all surface chemistries that could participate in hydrogen-bonding interactions with the incoming lignin, while PDADMAC cannot, we propose that the adsorption of lignin to the AuNPs is being driven by van der Waals forces under the conditions studied here. Since the Cit, PAH, and PAA-AuNPs all give comparable *K*_a_ values in this study, it is possible that the three capping agents are interacting with lignin through a common dominant intermolecular force, and hydrogen bonding is likely the most dominant intermolecular force that could occur between these AuNP surfaces and the phenols and carboxylic acids present in lignin. Since PDADMAC is the most hydrophobic functional group displayed by the AuNPs in our library, and lignin’s most abundant functional groups contain phenolic aromatic rings, we propose that hydrophobic interactions are the main driving force for lignin adsorption onto these AuNP surfaces. While dispersion forces are weaker than hydrogen bonds on a per-interaction basis (∼4 kJ/mol and ∼40 kJ/mol at the upper end, respectively), the number of hydrophobic interactions that lignin could achieve with the PDADMAC-AuNP surface could still lead to stronger binding overall. Of course, as with proteins, a variety of intermolecular interactions are likely mediating the adsorption of the lignin to the AuNP surfaces, and some combination of van der Waals forces and Coulombic interactions are likely in play when lignin binds to each of these surface chemistries. It would be informative to repeat this FQT experiment in a salt solution with higher ionic strength to see if the *K*_a_ values change for any of the AuNP surface chemistries; this would help confirm to what extent Coulombic forces may be mediating lignin binding. Unlike the QCM-D studies mention above, which were performed using carboxylate-modified lignin, it appears that electrostatics are not a primary driving force in unmodified lignin adsorption to these PE surfaces.^54–56^

The *K*_a_ values determined by FQT in this study are reasonably in line (and comparable in strength) to the affinity constants determined for serum proteins (like albumin) binding to functionalized AuNP surfaces.^25,26^ The *K*_a_ values obtained here for lignin–AuNP binding are 10–100 times larger than *K*_a_ values that have previously been obtained for bovine serum albumin binding to the surface of PAH-gold nanorods (*K*_a_ = 17.1 nM^–1^) and very comparable to *K*_a_ values obtained for α-synuclein binding to 90 nm Cit-AuNPs (*K*_a_ = 35 nM^–1^) obtained using FQT studies.^25,26^ Given the large size of the lignin polymer strands relative to the AuNP surfaces tested here, it seems reasonable that the many hydrophobic interactions that could act between the PE on the AuNP surface would lead to a larger affinity constant than individual proteins binding to an AuNP surface of a similar size.

Unlike the *K*_a_ measurements collected on protein-AuNP binding systems, the polydisperse nature of the lignin polymers complicates our understanding of the binding interactions between the lignin polymers and the AuNP surface. To better understand the nature of the lignin–AuNP interactions studied here, and the structure of the lignin–AuNP conjugates, it would be desirable to fractionate the lignin, and isolate fractions of lignin that are deliberately more comparable in size to (or significantly larger than) the AuNPs under study. In several recent studies of NOM fluorescence and NOM-AuNP conjugate formation, the NOM has been deliberately fractionated prior to initiating the binding study.^50–52,57,58^ Ultrafiltration (or tangential flow filtration) are two likely methods that could be used to isolate the smaller lignin polymers from our unfractionated kraft lignin.^36,57,58^ It would be potentially informative to see whether the different fractions of kraft lignin have comparable fluorescence, and whether they different fractions had similar binding affinities for our AuNP surfaces.

### Lignin–AuNP Affinity Constants Measurement by UV–vis Spectrophotometric Titration

In order to establish a comparative framework for the affinity constants determined by the FQT studies, UV–vis absorbance spectrophotometric titration was attempted to validate the binding interaction between the AuNP surface chemistries and lignin. The approach is frequently employed in spectroscopic determinations of affinity constants for proteins binding to AuNP surfaces.^25,26^ To determine the affinity constant (*K*_a_), the SPR absorbance wavelength (λ_max_) of each AuNP was monitored as the AuNPs were exposed to increasing concentrations of lignin solution (0–0.075 mg/mL, [AuNP] = 0.0033 nM, pH = 7.4). The λ_max_ was measured at room temperature after incubation for 60 min (Figures S6 and S10, Supporting Information). A gradual red shift in the SPR absorption maxima of AuNPs as the lignin concentration increases indicates an induced change in the dielectric environment around the AuNP surface. The observed plasmon response shift is then fitted to a Langmuir isotherm (Supporting Information). The slope of a linear Langmuir isotherm plot can also be used to determine the affinity constant.^25,26^ In our study, however, the absorption spectroscopy titration was not successful for these AuNP–lignin systems. In this study, there was no observable absorbance plasmon maxima shift for the surface chemistries except for Cit-AuNPs (Figures S6A and S10A). This implies that the wavelength of the absorption maxima is not altered due to lignin–AuNP binding, depending on the AuNP surface chemistry. This lack of sensitivity to lignin binding in SPR λ_max_ is most likely due to two possible causes: the dielectric constant of the particle surface does not change appreciably upon lignin binding, or the Langmuir isotherm (which assumes site-by-site monolayer binding of the corona former to the AuNP surface) is not appropriate to describe the adsorption of lignin to the AuNP surface. Since the majority of AuNP surface chemistries investigated here showed no significant shift in the λ_spr_ upon lignin binding, the absorbance spectroscopy titration data and alternative binding models were not further explored to obtain *K*_a_ values that could be used as a point of comparison for the *K*_a_ values obtained in the fluorescence quenching titration experiments.

## Conclusion

In this study, 90 nm citrate-stabilized gold nanoparticles were synthesized using a seeded growth approach. Following purification, the AuNP surfaces were functionalized with three polyelectrolytes (PAH, PAA, and PDADMAC) in a layer-by-layer wrapping approach, providing a library of nanoscale polymer surfaces with varying surface charges. The purified AuNPs were characterized by UV–vis absorbance spectroscopy, SEM, dynamic light scattering, ζ-potential analysis and FTIR spectroscopy. Characterization confirmed that the core size of the AuNPs was indeed just under 90 nm, and the particles were successfully functionalized with the desired polyelectrolytes. The affinity constants (*K*_a_) of each AuNP surface chemistry for kraft lignin were determined using fluorescence quenching titration, and the size and surface charge of the AuNP–lignin conjugates were characterized using DLS and ζ-potential analysis.

Three of the AuNP surface chemistries (Cit-(*K*_a_ = 92 ± 11 nM^–1^), PAH-(*K*_a_ = 107 ± 13 nM^–1^), and PAA-AuNPs (*K*_a_ = 87 ± 8 nM^–1^)) showed statistically indistinguishable affinity constants for lignin. In contrast, PDADMAC-AuNPs showed a significantly higher affinity constant (*K*_a_ = 240 ± 13 nM^–1^) for kraft lignin than the other surface chemistries tested, as determined by FQT analysis. This result suggests that (despite the fact that lignin is negatively charged under the pH regime studied) electrostatic adsorption may not be the primary driving force in lignin binding to nanoscale PE surfaces. Instead, weaker van der Waals forces (on a per-interaction basis), such as hydrophobic interactions, may be a stronger contributor in lignin adsorption to these polyelectrolyte surfaces. DLS and ζ-potential analysis further indicate that the structure of the lignin–AuNP conjugates differs based on the original surface charge of the AuNPs, and that lignin may not simply overcoat the surface of the NP as it absorbs. Instead, lignin may interact with the NP’s capping agent (possibly loosening the layers of PE, as indicated here with the PAA-AuNPs), or may promote hetero aggregation of the NPs with the incoming lignin (as was likely observed with the PDADMAC-AuNPs). It is important to note that (depending on the sizes of the NOM encountered by the ENMs), eco-coronas may have more complexity in size, charge and shape than would typically be observed in protein coronas on particles of similar sizes.

One of the main challenges in the use of FQT to measure the binding of kraft lignin to AuNP surfaces is that the lignin is not monodisperse and varies in polymer size and composition. The binding affinity of kraft lignin for the AuNP surfaces can be further elucidated by fractionating the lignin and determining whether the different lignin fractions have different affinities for the AuNP surfaces. Further fractionation of the lignin is likely necessary to really enable a fair comparison of how strongly different lignin components adsorb to these nanoscale surfaces. Lastly, the ability to functionalize AuNPs with charged polymers (while maintaining the fundamental optical properties of the AuNPs) may make them an appealing model system to study the affinity of eco-corona formers for nanoscale polymer surfaces across many different systems.

## Experimental Section

### Materials

Milli-Q deionized water (18 MΩ) was used throughout the study as a solvent for the preparation of all stock solutions. Hydrogen tetrachloroaurate trihydrate (HAuCl_4_·3H_2_O), hydroquinone (>99%), sodium chloride (>99%), sodium citrate tribasic dihydrate, and poly­(diallyldimethylammonium chloride) (*M*_w_ < 100,000, 35 wt % in H_2_O), poly­(allylamine hydrochloride) (*M*_w_ ∼ 17,500 GPC vs PEG std.), and poly­(acrylic acid, sodium salt) (*M*_w_ ∼ 8,000, 45 wt % in H_2_O) were obtained from Sigma-Aldrich, and used as received. Kraft Lignin, alkali (CAS: 8068-05-1, pH 6.5) was obtained from Sigma-Aldrich and was used after purification as follows. Powdered lignin was weighed out and dissolved in water using both gentle heating and sonication to give 500 mL of a solution with a total lignin concentration of 0.2 mg/mL. To remove any insoluble fraction of the lignin, the resultant solution was filtered using Whatman #1 folded filter paper and the filtrate solution was stored in the dark at room temperature for all subsequent binding studies. As a result, anywhere that a lignin concentration is subsequently indicated in this study, that lignin concentration is based on the original mass of the lignin added to the solution.

### Instrumentation

Absorbance spectroscopy was performed using an Agilent 8543 UV–visible spectrophotometer with a 1 cm standard quartz cuvette obtained from VWR. Fluorescence spectroscopy was performed on a Horiba FluoroMax-4 spectrofluorometer with Alpha Nanotech quartz cuvettes (1 cm path length). Particle size analysis was performed using the FEI Quanta 250 Field Emission Scanning Electron Microscope (SEM). SEM mounts and grids (Cu/SiO grids (PELCO)) were obtained from Ted Pella. Dynamic Light Scattering (DLS) and ζ-potential were performed using a Nanotrac Wave II/Zeta analyzer. Infrared spectroscopy was performed using an Alpha-P Fourier Transform Infrared spectrophotometer (FTIR) with a fixed Attenuated Total Reflectance (ATR) head accessory.

### Gold Nanoparticle (AuNP) Synthesis

90 nm Cit-AuNPs were prepared by previously published seeded growth synthesis methods. A gold nanoparticle seed solution was prepared by the standard citrate reduction method.^45^ Before synthesis, all glassware and stir bars were cleaned with *aqua regia* (**Caution**: Corrosive! Oxidizer!). Briefly, 30 mL of Milli-Q water and 300 μL of 1% (w/v) gold­(III) chloride trihydrate (HAuCl_4_·3H_2_O) aqueous solution were added to a 250 mL Erlenmeyer flask charged with a Teflon stir bar. The reaction mixture was placed on a magnetic stirring plate and brought to a boil while stirring at a vortex. As soon as the solution started boiling, 900 μL of 1% (w/v) sodium citrate was added, leading to a slow color change from pale yellow to a deep red, indicating the formation of 12 nm Cit-AuNP seed particles.

The original citrate-stabilized seeds were grown into larger gold nanospheres using the hydroquinone method in 10 mL batches as described by Perrault and Chan.^45^ 7.5 mL of the already prepared 1% (w/v) gold­(III) chloride trihydrate solution was placed in five 1.6 mL microcentrifuge tubes and centrifuged at 14,000× rpm for 60 min to remove any reduced gold species. 100 μL of the centrifuged gold chloride solution was added to 9.5 mL of Milli-Q water in a 15 mL centrifuge tube. A 35 μL volume aliquot of the particle seeds was then added. The resulting solution was stirred rapidly at room temperature followed by the addition of 22 μL of a 1 wt % sodium citrate solution and 100 μL of 0.03 M hydroquinone. This caused a slow color change to a red-purple solution, indicating the formation of large AuNPs. Stirring was stopped 30 min after the color change appeared complete, and at this time, 50 μL of aqueous sodium citrate (1 wt %) solution was added to further stabilize the particles. The resulting AuNP dispersion was left to stand overnight and then purified by centrifugation (8500*g* for 14 min). The pelleted particles were then resuspended in Milli-Q water.

### Layer-by-Layer Polyelectrolyte Functionalization on AuNP Surfaces

Three distinct AuNP polyelectrolyte surface chemistries were prepared through layer-by-layer (LBL) polyelectrolyte wrapping of the 90 nm citrate-stabilized gold nanoparticle dispersion, according to previously published methods.^46^ In this study, the original 90 nm Cit-AuNPs, were wrapped with three polyelectrolytes: poly­(diallyldimethylammonium chloride) (PDADMAC), poly­(allylamine hydrochloride) (PAH), and poly­(acrylic acid) (PAA). Each polyelectrolyte layer is wrapped over the previous layer in a layer-by-layer manner, resulting in AuNPs that are wrapped with PE layers of alternating charge. For the first polyelectrolyte layer (PAH-AuNP), 8.4 mL of purified aqueous 90 nm Cit-AuNP dispersion was introduced into a 15 mL centrifuge tube and subsequently mixed with 2.4 mL of a 10 mg/mL PAH solution, 2.4 mL of Milli-Q water, and 1.2 mL of 0.1 M aqueous NaCl solution. The resulting mixture was gently shaken for 1 h on an electric Benchmark shaker, maintained at 100 rpm (rpm). After incubation, the mixture was centrifuged at 4000*g* for 30 min, then the supernatant was carefully removed. The pelleted AuNPs were resuspended in Milli-Q water and stored in the dark for subsequent characterization and/or further LBL wrapping. The same general procedure was repeated for the second and third layers, wrapping PAA over the PAH-AuNP particles and wrapping PDAMAC over the PAA-AuNP particles, respectively.

### AuNP Characterization

The resulting functionalized AuNPs were characterized using several complementary techniques. The AuNP size and AuNP concentration were determined via UV–vis absorbance spectroscopy according to established methods.^43^ The maximum absorption wavelength (λ_spr_) of the AuNP dispersion was determined from the UV/visible absorbance spectrum using the equation below

1 $$d=\frac{\ln\left(\frac{\lambda_{spr}-\lambda_{0}}{L_{1}}\right)}{L_{2}}$$

Here, *d* is the diameter of the spherical AuNP. λ_0_, *L*_1_, and *L*_2_ have values of 512 nm, 6.53, and 0.0216 respectively.^43^

Imaging and formal core size analysis of the synthesized gold nanoparticle was performed using scanning electron microscopy (SEM). To image the AuNPs, an aqueous solution of the purified AuNP dispersion was drop cast onto the 1.5 mm Cu/SiO grids (PELCO) placed on pin stubs and allowed to air-dry at room temperature. SEM images of the particles were recorded at various magnifications and were used to determine the density of the synthesized gold nanoparticles by measuring the diameter of *n* ∼ 200 individual particles dispersed evenly in the image using the ImageJ (FIJI) software. The hydrodynamic diameter (*D*_h_) of the synthesized AuNP was measured by the dynamic light scattering. The hydrodynamic diameter (*D*_h_) was measured at a [AuNP] = 0.0033 nM, in bicarbonate-buffered Milli-Q water, pH = 7.4.

ATR-FTIR spectra of the AuNP samples were obtained to verify polyelectrolyte functionalization of the AuNPs. Prior to analysis, purified AuNP dispersions were digested with the addition of molecular iodine, and then the digested solution was separated by centrifugation (5000*g*, 14 min). The supernatant was then removed and was analyzed by ATR-FITR to determine which polyelectrolytes were bound to the AuNP surface. FTIR spectra were recorded in the transmission mode between 400 cm^–1^ and 4000 cm^–1^, with 24 scans collected on each sample. To verify the surface charges of the functionalized particles, the ζ-potential of each functionalized AuNP dispersion was analyzed. The ζ-potential was measured at a [AuNP] = 0.0033 nM, in bicarbonate-buffered Milli-Q water, pH = 7.4.

### Time-Dependent and Concentration-Dependent Lignin Incubation Studies

In order to determine how long an incubation period was required to produce a stable lignin–AuNP conjugate, 700 μL of previously purified AuNP solution was introduced into 1.5 mL microcentrifuge tubes followed by 10 μL of 0.1 M bicarbonate buffer, and 400 μL of 0.35 mg/mL lignin solution. UV–visible absorbance spectra of the mixture were taken immediately after thoroughly mixing the components at 0 min and further scans were taken at 10, 20, 30, 45, 50, 60, 70, 80, and 90 min.

In order to determine how the physicochemical properties of the lignin–AuNP conjugates change with increasing lignin concentration, 300 μL of 0.0142 nM of aqueous AuNP solution were combined with 550 μL of Milli-Q water and mixed with 0.2 mg/mL lignin stock solution in varying concentrations from 0 to 0.075 mg/mL in a 1.5 mL microcentrifuge tube. Each tube was covered, gently vortexed, labeled, and incubated in the dark at room temperature for 60 min. After a 60 min incubation period, UV–vis absorbance spectra were recorded in a 1 cm quartz cuvette. UV–visible absorbance spectra were recorded at room temperature. The experiment was run in triplicate at each lignin concentration. Corresponding DLS and ζ-potential measurements were obtained under the same incubation conditions to determine the hydrodynamic diameter (*D*_h_) and surface charge for the AuNPs pre- and postlignin incubation. Polydispersity index (PDI) values were obtained from the DLS data post-analysis by taking the variance of the peak width and then dividing by the square of the average *D*_h_ value. The pH of all lignin–AuNP solutions were stabilized by the addition of 10 μL of 1.0 M bicarbonate buffer (pH = 7.4).

### Affinity Constants Measurement by Fluorescence Quenching Titration

The fluorescence emission of lignin was measured at a constant concentration (0.2 mg/mL) in the presence of an increasing concentration of citrate-AuNP, PAH-AuNP, PAA-AuNP, and PDADMAC-AuNP ([AuNP] = 0–0.0033 nM in particles). Ultrapure Milli-Q water was used as a solvent and the AuNP–lignin mixtures were incubated in 1.5 mL microfuge tubes (dark at room temperature, *T* = 25 °C) for 60 min to ensure the formation of a stable lignin–AuNP conjugate. The pH of the lignin–AuNP solutions were stabilized by the addition of 10 μL of 1.0 M bicarbonate buffer (pH = 7.4). After incubation, fluorescence spectra of the lignin–AuNP solutions were recorded using a Horiba FluoroMax-4 fluorescence spectrofluorometer with excitation at 290 nm and emission recorded from 300 to 500 nm. All fluorescence measurements were obtained in triplicate (*n* = 3).

Fluorescence intensity (counts per second) at 365 nm (λ_em_ max) was used to determine the fluorescence ratios (*F*_0_/*F*) in the presence of the AuNP quenchers, and Stern–Volmer plots were created by plotting fluorescence ratios against the AuNP concentrations.^25,26,30^ Here, *F*_*0*_ is the fluorescence intensity in the absence of AuNPs and *F* is the emission intensity at a specific concentration of AuNP. The emission intensity data obtained at 365 nm were fitted to the Stern–Volmer equation (eq 2), which relates the static fluorescence quenching of lignin to AuNP concentrations.

2 $$\frac{F_{0}}{F}=K_{sv}\left[AuNP\right]+1$$

If the AuNP forms a stable complex with the lignin, the Stern–Volmer quenching constant (*K*_sv_) is equivalent to the lignin–AuNP binding constant (*K*_a_).^25,26,30^

## Supplementary Material



## Notes

Detailed description of materials and methodologies as well as supplemental data (additional DLS data, FTIR spectral data, and UV–vis absorbance data) (PDF)
