# Supplementary material for: Surface Chemistry-Dependent Binding Interactions between Kraft Lignin and Polyelectrolyte-Encapsulated Gold Nanoparticles
Source: ACS Omega. 2025 Oct 27;10(44):52909–20. doi: 10.1021/acsomega.5c07312 (PMC12612877; doi:10.1021/acsomega.5c07312)
Supplement: Supplementary file 1 [file ao5c07312_si_001.pdf]

Supporting Information

Surface Chemistry-Dependent Binding Interactions between Kraft Lignin and Polyelectrolyte-Encapsulated Gold Nanoparticles

*Akinsola A. Oluwaseun, Samuel E. Lohse**

Central Washington University, Department of Chemistry, 400 E University Way, Ellensburg, WA, 98926.

E-mail: Samuel.lohse@cwu.edu

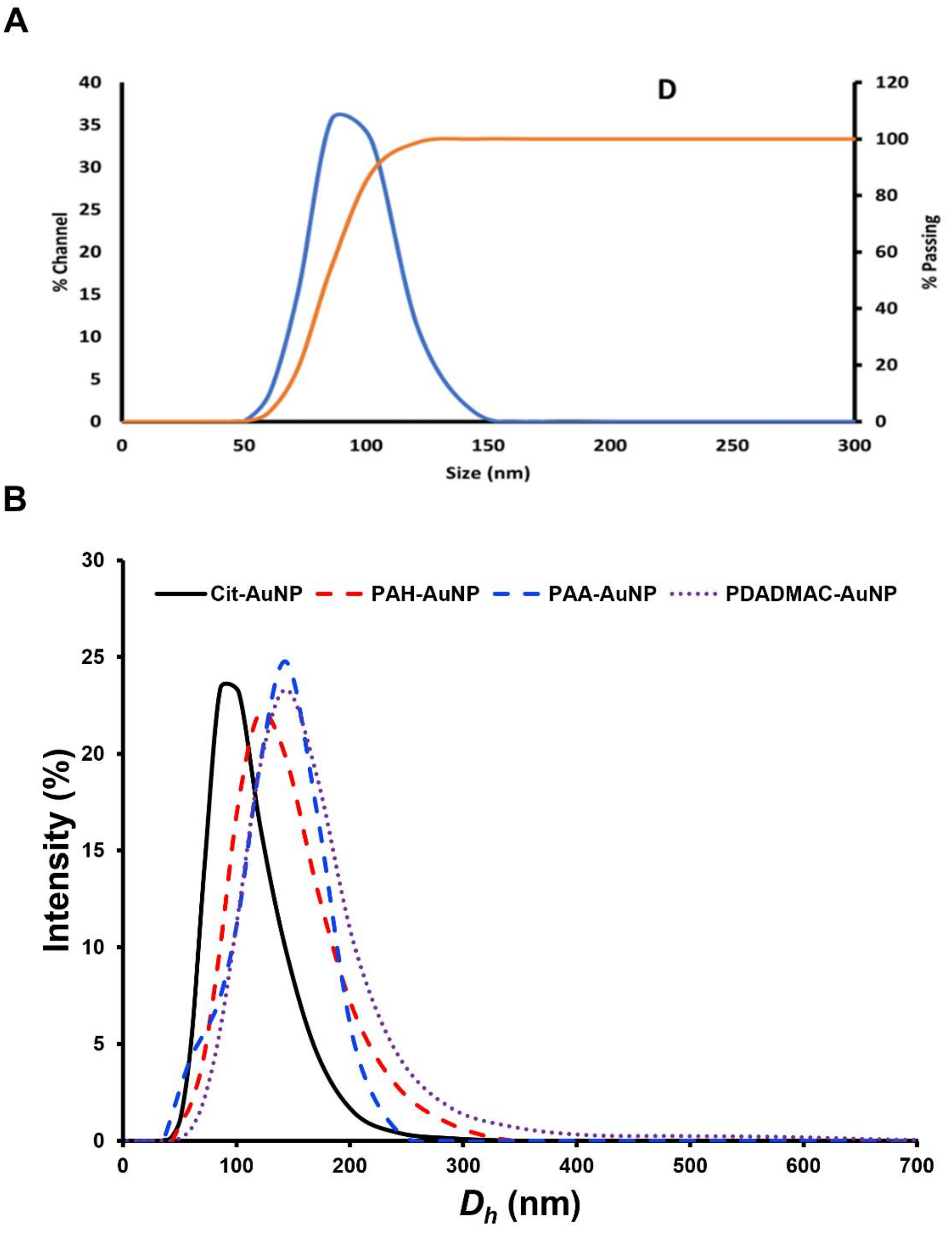

Figure S1. (A) DLS particle size distribution plot indicating the size range of hydrodynamic diameters (D_h) (% channel, blue trace) and the cumulative percentage of particles below and above each distribution band (% passing, orange trace) for purified Cit-AuNP dispersions. (B) Dynamic light scattering intensity particle size distribution plot indicating the distribution of hydrodynamic diameters for purified AuNP dispersions. DLS measurements obtained at pH = 7.4 (1 mM bicarbonate buffer), [AuNP] = 0.0033 nM. No detectable peaks D_h > 700 nm were observed in any sample in the DLS analysis.

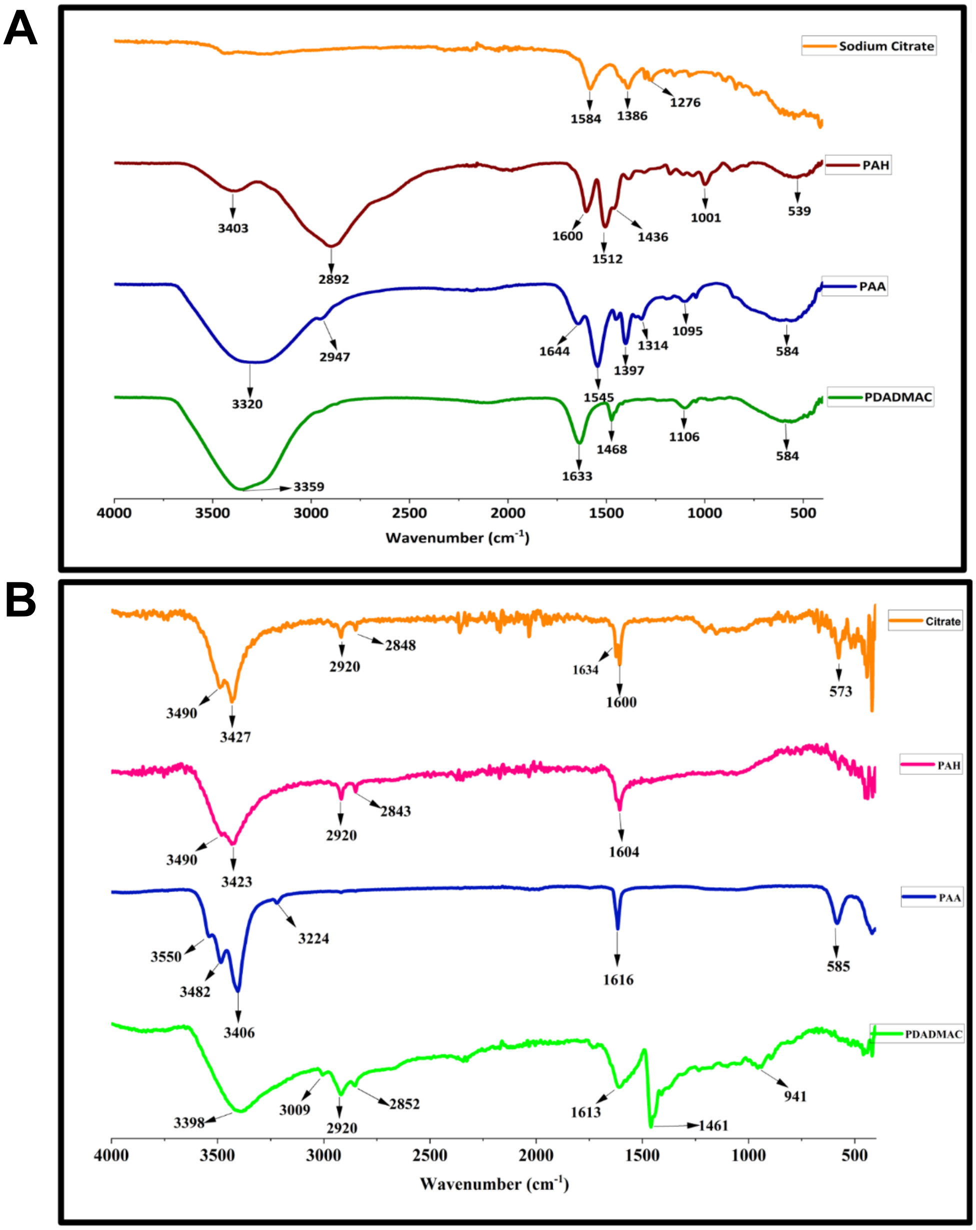

Figure S2. FTIR Spectra of: (A) individual capping agents, including sodium citrate (orange), PAH (maroon), PAA (blue), and PDADMAC (green), and (B) FTIR spectra of iodine-digested citrate and PE-coated AuNPs.

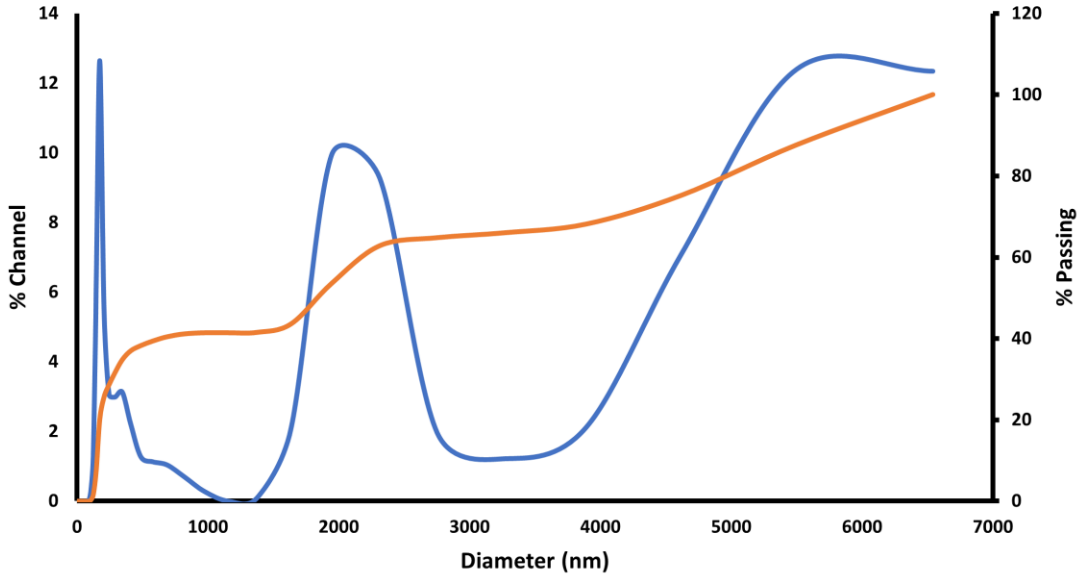

Figure S3. DLS size distribution plot indicating the size range of lignin in solution (% channel, blue trace) and the cumulative percentage of particles below and above each distribution band (% passing, orange trace).

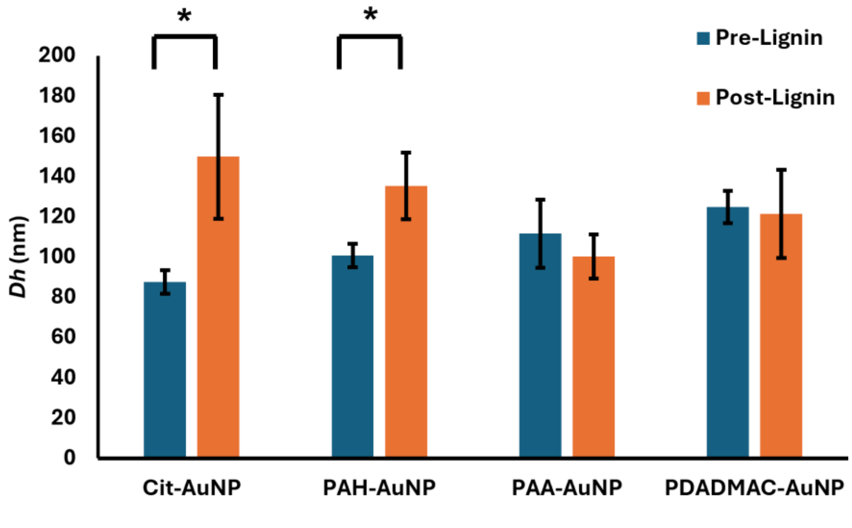

Figure S4. Comparison of the average hydrodynamic diameters (D_h) of AuNP surface chemistries pre- and post-lignin exposure. Incubated at 25 °C with 0.0033 nM concentration of AuNPs in 0.2 mg/mL lignin, pH = 7.4 (1 mM bicarbonate buffer). *- indicates that the D_h is statistically different pre- and post-lignin exposure ($p < 0.05$) for this AuNP surface chemistry.

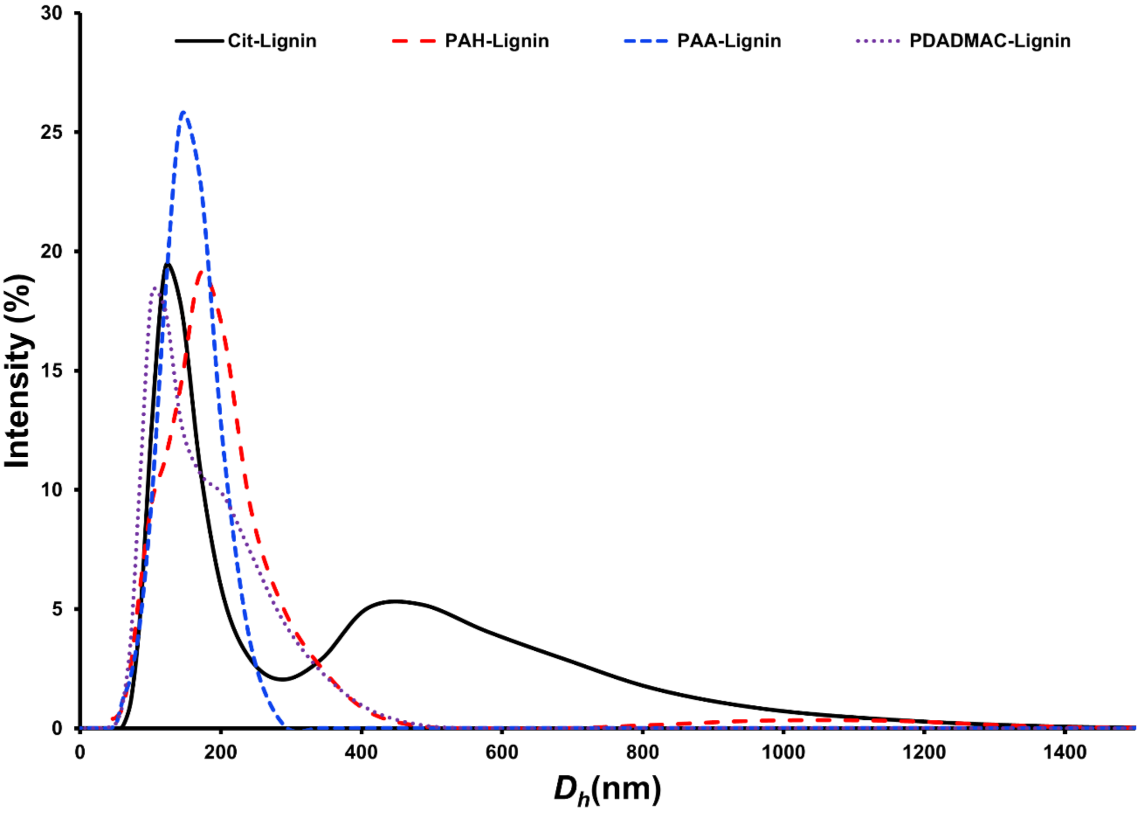

Figure S5. Dynamic light scattering intensity particle size distribution plot indicating the distribution of hydrodynamic diameters (D_h) for lignin-AuNP conjugates. Cit-(black, solid line), PAH-(red dashed line), PAA-(blue dashed line), and PDADMAC-(purple dotted line) AuNP dispersions. DLS measurements obtained at pH = 7.4 (1 mM bicarbonate buffer), [AuNP] = 0.0033 nM, [lignin] = 0.2 mg/mL. No detectable peaks D_h > 1400 nm were observed in any sample in the DLS analysis.

Table S1. Polydispersity Index (PDI) Values for Different AuNP Surface Chemistries and Corresponding Lignin-AuNP Conjugates

AuNP Surface Chemistry	*PDI Pre-Lignin Exposure**	*PDI Post-Lignin Exposure*
Cit	0.004	0.042
PAH	0.003	0.015
PAA	0.05	0.012
PDADMAC	0.004	0.03

**Pre-Lignin exposure conditions are Milli-Q water at pH = 7.4 (bicarbonate buffer), [AuNP] = 0.0033 nM. Post-Lignin exposure conditions are AuNP-lignin conjugates prepared in 0.2 mg/mL lignin solution at pH = 7.4 (1 mM bicarbonate buffer), [AuNP] = 0.0033 nM. PDI =* $s^2/(\text{mean } D_h)^2$ *for the primary particle peak.*

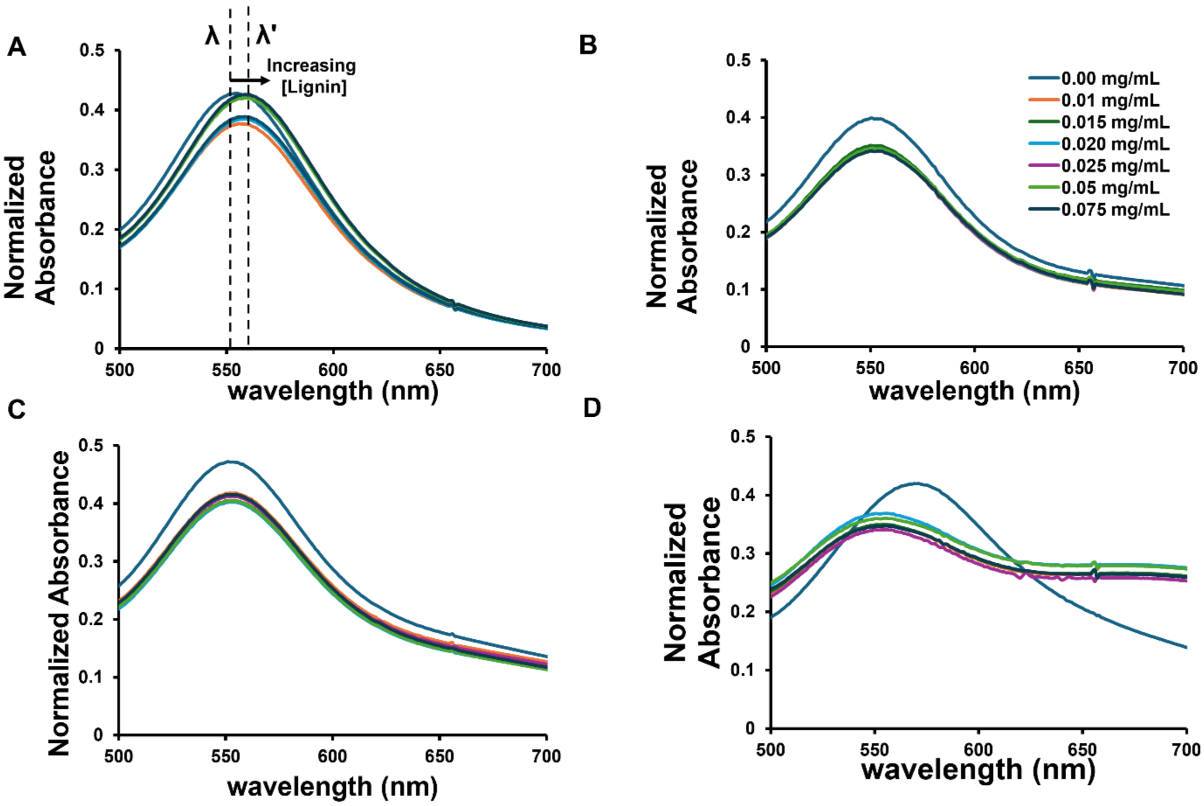

Figure S6. UV-vis absorbance spectra of (A) Cit-AuNP, (B) PAH-AuNP, (C) PAA-AuNP, and (D) PDADMAC-AuNPs incubated in increasing [lignin] (0-0.075 mg/mL). Spectra obtained at [AuNP] = 0.0033 nM, pH = 7.4 (1 mM bicarbonate buffer).

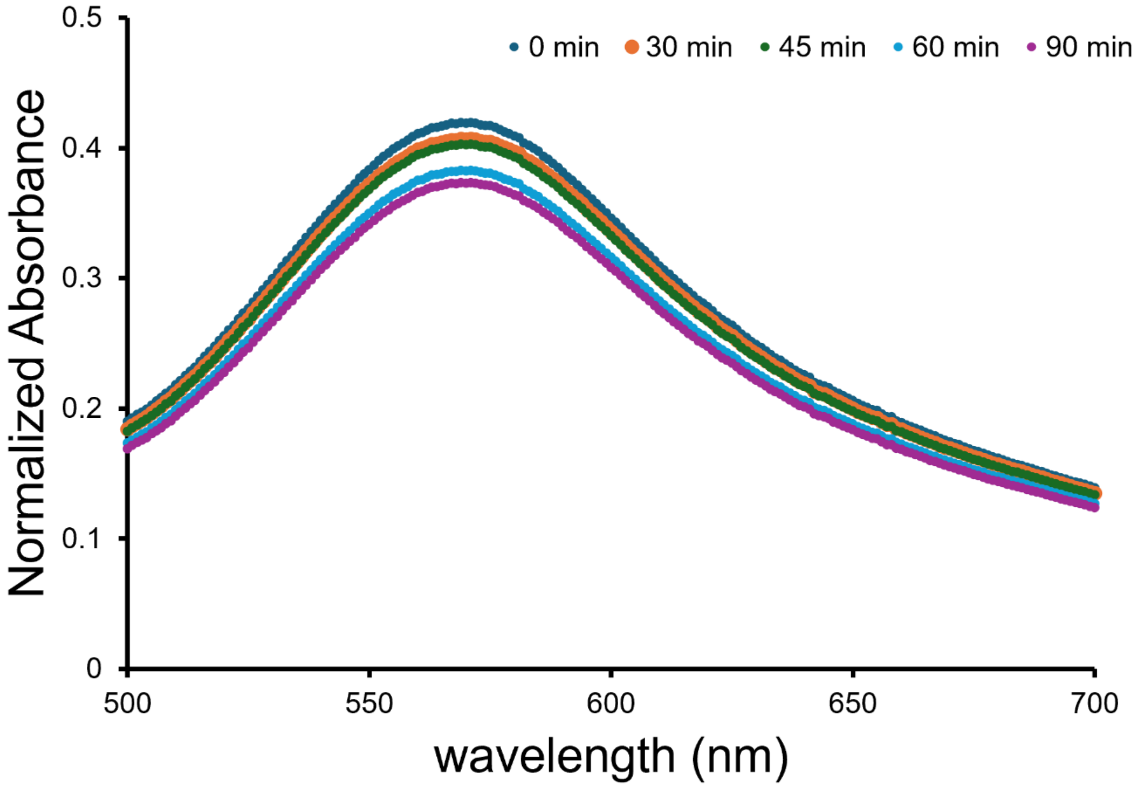

Figure S7. UV-vis absorbance spectra of PDADMAC-AuNPs incubated for 90 min under fluorescence quenching exposure conditions (0.2 mg/mL lignin). Spectra obtained at [AuNP] = 0.0033 nM, pH = 7.4 (1 mM bicarbonate buffer).

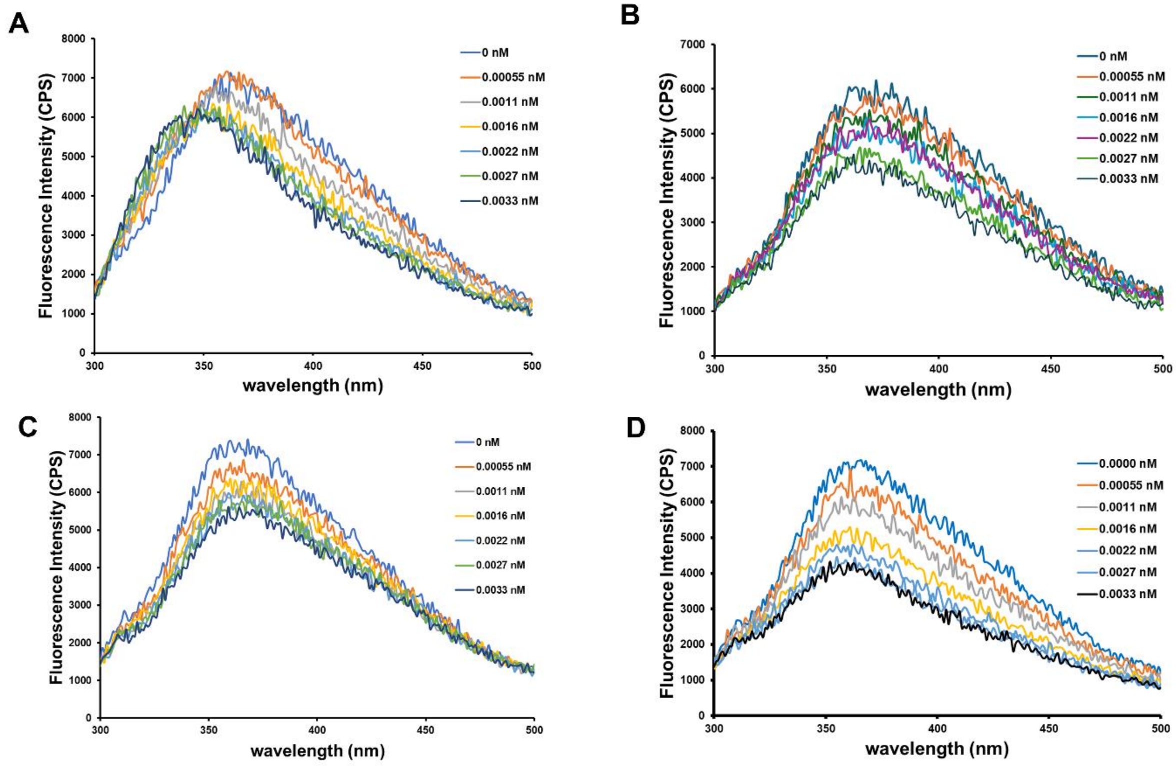

Figure S8. Fluorescence emission spectra of 0.2 mg/mL lignin incubated for 60 minutes in the dark at 25 °C with 0 to 0.0033 nM concentrations of (A) Cit-AuNPs, (B) PAH-AuNPs, (C) PAA-AuNP, and (D) PDADMAC-AuNP, in Milli-Q water, pH = 7.4 (1 mM bicarbonate buffer).

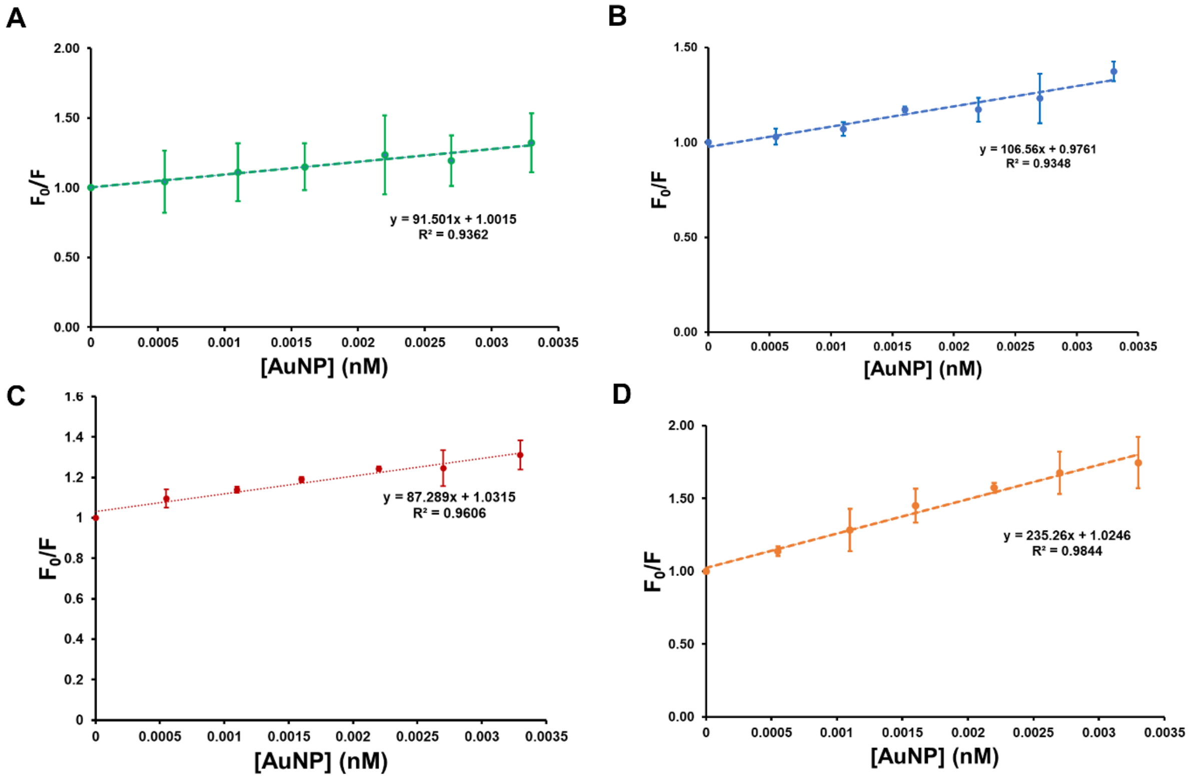

Figure S9. Stern-Volmer plots of 0.2 mg/mL lignin incubated for 60 minutes in the dark at 25 °C with 0-0.0033 nM concentrations of (A) Cit-AuNPs, (B) PAH-AuNPs, (C) PAA-AuNP, and (D) PDADMAC-AuNP, in Milli-Q water, pH = 7.4 (1 mM bicarbonate buffer). Error bars shown are the standard deviation based on three replicate measurements (n = 3).

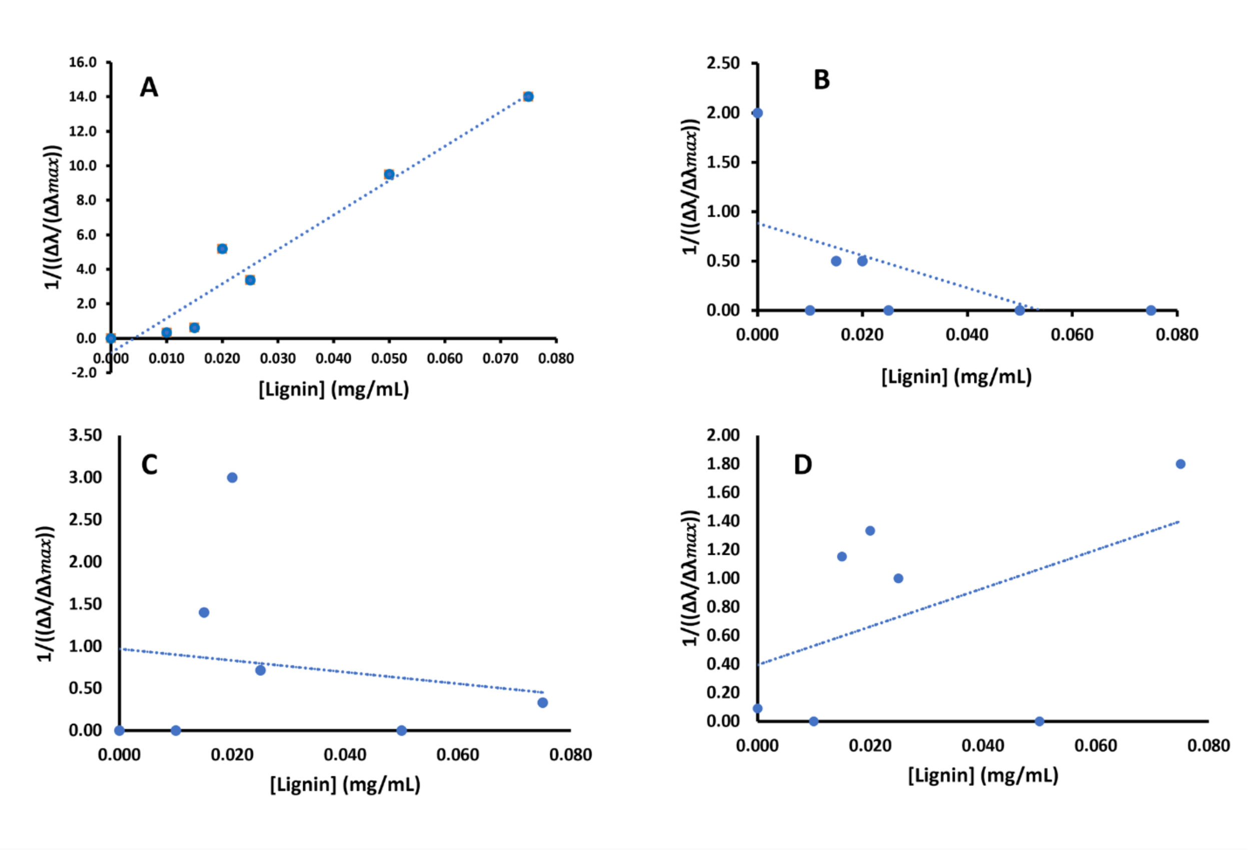

Figure S10. Langmuir adsorption isotherm plots of (A) Cit-AuNP, (B) PAH-AuNP, (C) PAA-AuNP and (D) PDADMAC-AuNP, added to an increasing lignin concentration (0 – 0.075 mg/mL) in Milli-Q water. Spectra obtained at [AuNP] = 0.0033 nM, pH = 7.4 (1 mM bicarbonate buffer). Error bars are shown, but in many cases, do not extend beyond the width of the data points. Error bars represent the standard deviation based on three replicate measurements (n = 3).

Lignin-AuNP Affinity Constants Measurement by UV-Vis Spectrophotometric Titration. 300 µL of 0.0142 nM of citrate-AuNP, PAH-AuNP, PAA-AuNP, and PDADMAC-AuNP were combined with 500 µL of Milli-Q water, 50 µL of 0.1 M bicarbonate buffer, and mixed with 0.2 mg/mL aqueous lignin solution to give lignin solutions with varying concentrations of 0-0.075 mg/mL and a constant [AuNP] = 0.005 nM. Each tube was covered, gently vortexed, labeled, and incubated in the dark at room temperature (25 °C) for 60 minutes. After a 60-minute incubation period, UV-vis absorbance spectra were recorded from 400-800 nm. The experiment was performed in triplicate.

The wavelength at which the maximum absorbance was observed (λ_{max}) for each analysis was used to determine the shift in the plasmon peak position ratios ($\Delta\lambda/\Delta\lambda_{max}$). According to the equation below, the Langmuir adsorption isotherm model was used to determine the binding constant between AuNP and Lignin interaction.

$$\frac{\Delta\lambda}{\Delta\lambda_{\mathrm{max}}} = \frac{K_a[\mathrm{Q}]}{1 + K_a[\mathrm{Q}]} \qquad \text{(S1)}$$

Here, $\Delta\lambda$ and $\Delta\lambda_{max}$ are the change and maximum change in the plasmon peak wavelength of the molecule of interest, K_a is the binding constant, and [Q] is the lignin concentration for each AuNP-Lignin measurement point.[38] Langmuir adsorption isotherm plots were generated by plotting $\frac{1}{\left(\frac{\Delta\lambda}{\Delta\lambda_{max}}\right)}$ against the concentration of lignin in mg/mL.
